# Hearing Outcome and Predictors after Implanting Bone Conduction or Middle Ear Implants in Ears with Refractory Otitis Media

**DOI:** 10.3390/jcm12124086

**Published:** 2023-06-16

**Authors:** Sho Kurihara, Takeshi Nakamura, Kan Kubuki, Hiroyuki Koga, Takashi Goto, Shouken Shimoara, Akira Ganaha, Yuusuke Yamada, Kuniyuki Takahashi, Tetsuya Tono

**Affiliations:** 1 Department of Otorhinolaryngology, Head and Neck Surgery, University of Miyazaki, Miyazaki 889-2155, Japan; takeshi_nakamura@med.miyazaki-u.ac.jp (T.N.); kan_kubuki@med.miyazaki-u.ac.jp (K.K.); hiroyuki_koga@med.miyazaki-u.ac.jp (H.K.); takashi_goto@med.miyazaki-u.ac.jp (T.G.); ganaha.akira.t8@iuhw.ac.jp (A.G.); yuusuke_yamada@med.miyazaki-u.ac.jp (Y.Y.); kuniyuki_takahashi@med.miyazaki-u.ac.jp (K.T.); tono@med.miyazaki-u.ac.jp (T.T.); 2 Department of Otorhinolaryngology, Head and Neck Surgery, The Jikei University School of Medicine, Minato-ku 105-8461, Japan; 3 Miyazaki University Hospital Hearing Care Center, Miyazaki 889-1601, Japan; shouken_shimoara@med.miyazaki-u.ac.jp; 4 Department of Otorhinolaryngology, Head and Neck Surgery, International University of Health and Welfare Narita Hospital, Narita 286-0124, Japan; 5 Department of Otorhinolaryngology, Head and Neck Surgery, University of Ryukyus, Nakagami-gun 903-0125, Japan; 6 Department of Otorhinolaryngology, Head and Neck Surgery, International University of Health and Welfare Hospital, Nasushiobara 329-2763, Japan

**Keywords:** bone conductive implant, middle ear implant, refractory otitis media, Baha, Vibrant Soundbridge, conductive hearing loss, mixed hearing loss

## Abstract

Bone conduction implants (BCIs) and middle ear implants (MEIs) are promising options for individuals with persistent chronic inflammation of the middle or outer ear. However, the structure of the middle ear is often altered in patients who undergo mastoidectomy or posterior wall removal for refractory otitis media, leaving uncertainty regarding the efficacy of hearing devices. Only a few studies have examined auditory outcomes based on the etiology of hearing impairment. We investigated hearing outcomes, including speech audiometry, in patients who underwent implantation after surgery for refractory otitis media. Our findings indicated that patients who received BCIs or MEIs achieved favorable hearing outcomes. Furthermore, a correlation was observed between the preoperative bone-conduction threshold at 1 kHz in the better ear and the sound-field threshold at 1 kHz with BCIs, whereas no correlation was observed between the preoperative bone-conduction threshold and the sound-field threshold with MEIs. This study highlights the positive impact of BCIs and MEIs in patients who undergo implantation after surgery for refractory otitis media. Additionally, our study identified parameters that predict postoperative efficacy.

## 1. Introduction

When the improvement in auditory function remains inadequate following conventional surgical interventions, such as tympanoplasty or tympano-revisions for refractory otitis media, the prevailing course of action is to employ a conventional hearing aid that operates via air conduction. Nevertheless, it is well established that the utilization of air-conduction (AC) hearing aids can pose challenges in cases of persistent chronic inflammations that impact the outer ear canal/radical cavity. In such scenarios, bone conduction implants (BCIs) and middle ear implants (MEIs) represent promising options for hearing restoration after considering non-surgical alternatives such as bone-conduction (BC) hearing aids and cartilage-conduction hearing aids [1]. BCIs and MEIs have been in development since the 1970s and have been widely implemented in patients with conductive or mixed hearing loss [2]. Multiple systematic reviews have investigated the effects of BCIs on patients with conductive or mixed hearing loss [3,4,5,6], and each review reported that BCIs enhance hearing function, as they all demonstrated improvements in hearing thresholds compared with no treatment. However, there is currently no definitive consensus regarding the enhancement of audiometric speech outcomes. There have also been various systematic reviews investigating the effects of MEIs on patients with conductive or mixed hearing loss [7,8,9], which revealed that MEIs can enhance hearing thresholds and result in clinically significant improvements in speech reception thresholds and word recognition compared with no treatment.

These findings provide useful information regarding the implantation of BCIs or MEIs in patients with conductive or mixed hearing loss. However, in postoperative patients with refractory otitis media, the structure of the middle ear is often altered by mastoidectomy or posterior wall removal, and it is unclear whether the hearing device is effective. Only a limited number of reports have investigated auditory outcomes based on the etiology of hearing impairment. Cooper et al. demonstrated that patients with chronic suppurative otitis media and those with congenital conductive hearing loss exhibited similar improvements in mean free-field warble-tone thresholds when using a bone-anchored hearing aid (BAHA) approach [10]. Henseler et al. also reported positive long-term outcomes of Vibrant Soundbridge (VSB; MED-EL, Innsbruck, Austria) implantation (floating mass transducer (FMT) within the round window niche) in 10 patients after subtotal petrosectomy [11]. Although these reports provide valuable insights into hearing compensation scenarios for postoperative patients with refractory otitis media, additional case studies are necessary to evaluate the hearing outcomes of these devices thoroughly.

Therefore, this study examined the hearing outcomes of patients who underwent implantation after surgery for refractory otitis media using speech audiometry. Additionally, preoperative parameters that may predict the effectiveness of hearing outcomes after implantation were investigated. These results have significant implications for the selection of appropriate hearing devices for postoperative patients with refractory otitis media and for predicting the effect of devices.

## 2. Materials and Methods

### 2.1. Participants

This study retrospectively analyzed 23 patients who underwent implantation of a Baha (Cochlear, Sydney, Australia) and 13 who underwent implantation of a VSB after surgery for refractory otitis media. Patients included in the study met the Japanese criteria [12,13], which requires a bone conduction hearing level of less than 45 dB HL for Baha5 and 55 dB HL for Baha5 POWER, as well as a bone conduction hearing level of less than 45 dB HL at 500 Hz, 50 dB HL at 1000 Hz, and 65 dB HL at both 2000 and 4000 Hz for VSB. These patients underwent surgery at the University of Miyazaki Hospital between 2012 and 2021. All patients had used their devices for at least 6 months. The characteristics and pure-tone thresholds for each patient are presented in Table 1 and Table 2. In one patient implanted with an MEI, an FMT had to be reimplanted 5 years after the initial surgery. This report used data from the initial surgery, during which the functional gain (FG) was measured correctly.

### 2.2. Fitting of BAHA

All 23 patients used the Baha Connect System. In total, 22 patients used the Baha 5 sound processor, and 1 patient used the Baha 5 Power sound processor fitted by experienced audiologists using Baha Fitting Software. The final gain settings were established by measuring the actual thresholds using the BC Direct function of the fitting software.

### 2.3. Fitting of VSB

The FMT was coupled to the round window in 12 patients and to the stapes in 1 patient. Fitting was performed by experienced audiologists, and the final gain settings were established by measuring the actual thresholds using a vibrogram.

### 2.4. Sound-Field Thresholds with Baha and VSB

The hearing threshold was measured using these devices. Inspection sounds were presented using audiometers (AA-H1; Rion, Tokyo, Japan) that met the Japanese industrial standards.

### 2.5. Word Recognition Test

To evaluate the word recognition score (WRS), the 67-S (Japanese nonsense monosyllable word-list) was evaluated 1 m from the front speaker unit in a shielded room. Answers were obtained by uttering a sound. All speech perception test grades were calculated as percentages of correct answers. Experienced audiologists performed all the tests.

### 2.6. Masking of the Contralateral Ear

The contralateral ear was covered with earmuffs and ear plugs or masked with narrowband noise during pure-tone and sound-field audiometry and with broadband noise during speech tests. Experienced audiologists determined the necessity of masking.

### 2.7. Statistical Analysis

The results were analyzed using GraphPad Prism 9 software (GraphPad Software, San Diego, CA, USA). The results are presented as mean and standard deviation. The level of significance was set at 5%. Two-group comparisons were performed using the two-tailed Wilcoxon signed-rank test. Associations between two variables were evaluated using Pearson’s correlation and linear regression.

## 3. Results

### 3.1. Baha: Pure-Tone Audiogram

**Figure 1:**
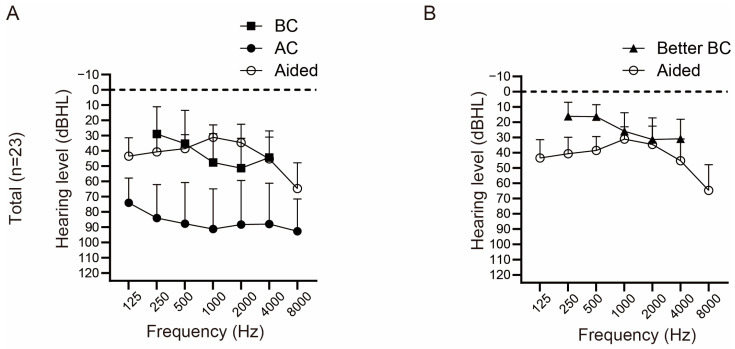
Average hearing and Baha-aided thresholds. (**A**) Mean air conduction (AC), bone conduction (BC) hearing thresholds, and sound field thresholds aided by Baha by masking the better-hearing ear (*n* = 23). (**B**) Mean BC threshold of the better-hearing ear is depicted together with Baha-aided thresholds (*n* = 23).

The preoperative pure-tone and aided hearing thresholds of all patients who underwent Baha implantation are shown in Figure 1A. The mean AC and BC thresholds at frequencies of 500, 1000, 2000, and 4000 Hz on the implanted side were 88.70 ± 25.07 dB HL and 44.62 ± 17.53 dB HL, respectively.

### 3.2. Baha: Sound-Field Thresholds

**Figure 2:**
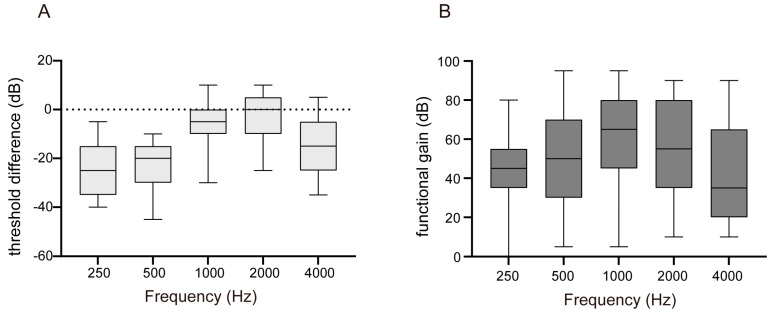
Hearing outcomes aided with Baha. (**A**) Difference between the better bone-conduction and aided threshold in each frequency. (**B**) Functional gain (FG) in each frequency. FG was calculated as the difference between the air-conduction threshold in the implanted side and the aided sound-field threshold.

Figure 1A,B illustrate the mean hearing thresholds in the sound field with Baha while masking the better-hearing ear. The mean thresholds were found to be 37.34 ± 8.36 dB HL. As it has been previously reported that aided postoperative sound-field thresholds are correlated with the BC threshold of the better ear [7], the BC thresholds in the better-hearing ear are depicted for comparison purposes. The mean BC threshold on the contralateral side was 31.47 ± 14.41 dB HL. The differences between the better BC and aided thresholds at each frequency are shown in Figure 2A. The results showed smaller differences at 1 and 2 kHz, whereas larger differences were observed at lower and higher frequencies. Notably, most Baha users do not use it for thresholds higher than the BC threshold.

### 3.3. Baha: Functional Gain

FG was calculated as the difference between the AC threshold on the implanted side and aided sound-field threshold. The results showed a particularly large FG at 1 kHz, with FG values at each frequency as follows: 250 Hz, 43.26 ± 19.52 dB HL; 500 Hz, 49.13 ± 25.66 dB HL; 1000 Hz, 60.00 ± 26.54 dB HL; 2000 Hz, 53.70 ± 25.59 dB HL; and 4000 Hz, 42.61 ± 24.30 dB HL (Figure 2B). The average FG value at the four frequencies was 51.36 ± 25.77 dB HL.

### 3.4. Baha: Correlation between Preoperative BC Threshold and Aided Threshold

**Figure 3:**
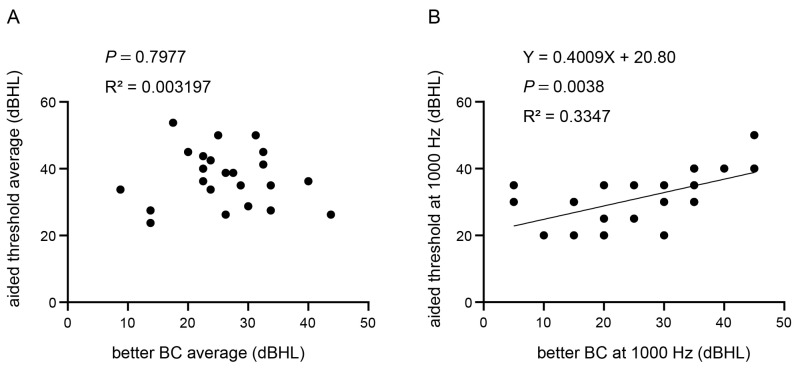
Correlation between preoperative bone-conduction (BC) threshold and aided threshold with Baha. (**A**) Mean BC thresholds at 500, 1000, 2000, and 4000 Hz for the better BC threshold were treated as independent variables, while the mean aided threshold was treated as the dependent variable. (**B**) Preoperative BC threshold at 1000 Hz for the better-hearing ear was used as the independent variable and the aided threshold at 1000 Hz was used as the dependent variable.

Linear regression analysis was conducted to examine the relationship between the preoperative BC threshold and aided threshold to investigate the potential of the preoperative BC threshold as a predictor of the efficacy of Baha. Specifically, the mean BC thresholds at 500, 1000, 2000, and 4000 Hz for the better BC threshold were treated as independent variables, whereas the mean aided threshold was treated as the dependent variable. However, the results revealed no significant correlation between these variables, as evidenced by an R^2^ value of 0.003 and a *p*-value of 0.798 (Figure 3A). Conversely, when the preoperative BC threshold at 1000 Hz for the better-hearing ear was used as the independent variable and the aided threshold at 1000 Hz was used as the dependent variable, a positive correlation was observed, with an R^2^ value of 0.335 and a *p*-value of 0.004 (Figure 3B).

### 3.5. Baha: Word Recognition Score

**Figure 4:**
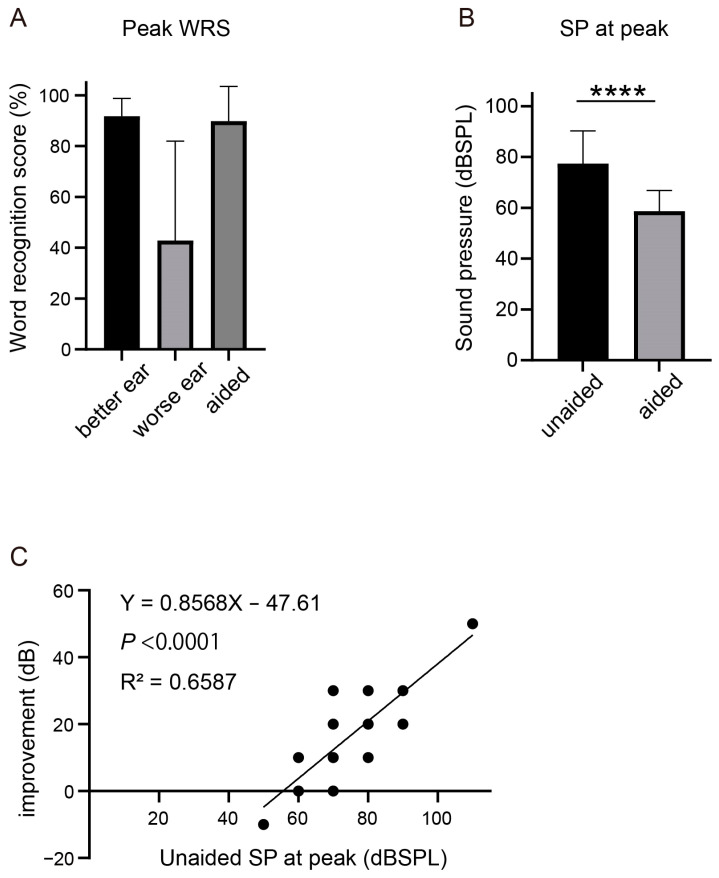
Word recognition score (WRS) compared between unaided and aided condition with Baha. (**A**) Peak WRS in unaided condition for both ears and when aided with Baha. (**B**) Sound pressure (SP) required to reach the peak WRS in unaided and aided condition. **** *p* < 0.0001. (**C**) Correlation between preoperative SP at peak and the improvement of SP at peak.

In each patient, the WRS was measured in the aided or unaided condition with varying sound pressures. For each patient, the sound pressure was increased until the WRS reached its peak. However, the peak WRS with Baha did not improve compared to that of the better ear preoperatively (Figure 4A). Interestingly, the sound pressure (SP) required to reach the peak WRS was significantly reduced by Baha (*p* < 0.001) (Figure 4B). To further investigate whether this improvement could have been predicted preoperatively, we used the preoperative SP at peak WRS as the independent variable and the improvement in SP at peak WRS as the dependent variable. Our analysis revealed a positive correlation between these variables, with an R^2^ value of 0.659 and a *p*-value < 0.001.

### 3.6. VSB: Pure-Tone Audiogram

**Figure 5:**
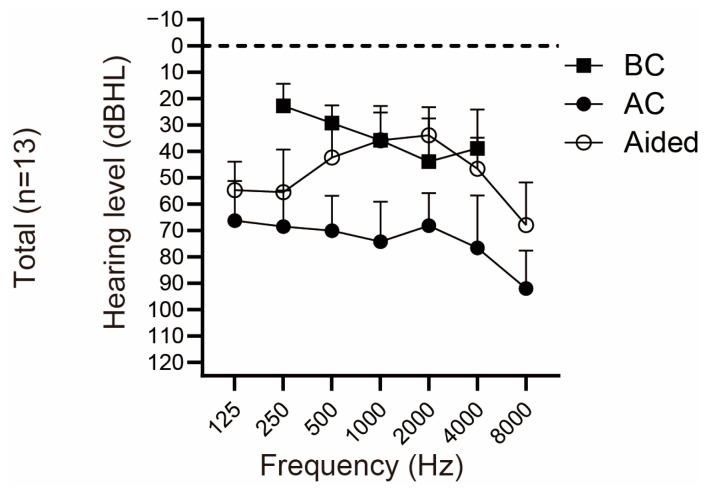
Average hearing and aided thresholds with Vibrant Soundbridge. Mean air-conduction (AC), bone-conduction (BC) hearing thresholds, and sound field thresholds aided by VSB by masking (*n* = 13).

The mean preoperative pure-tone audiogram and aided hearing threshold of all patients who underwent VSB implantation are shown in Figure 5. The average AC and BC thresholds at 500, 1000, 2000, and 4000 Hz on the implanted side were 72.21 ± 12.57 dB HL and 36.92 ± 11.45 dB HL, respectively.

### 3.7. VSB: Sound-Field Thresholds

**Figure 6:**
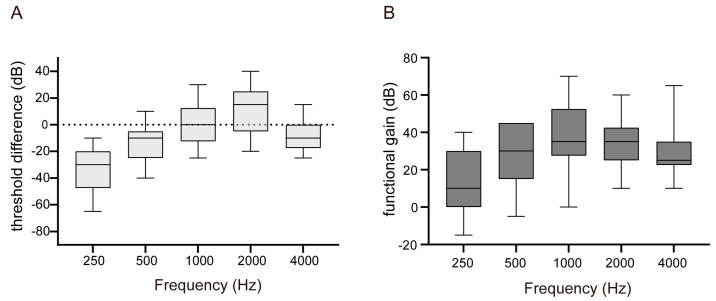
Hearing outcomes aided with Vibrant Soundbridge. (**A**) Difference between the bone-conduction and aided threshold in each frequency. (**B**) Functional gain (FG) in each frequency. FG was calculated as the difference between the air-conduction threshold in the implanted side and the aided sound-field threshold.

Figure 5 illustrates the average aided sound-field threshold with VSB while masking the better-hearing ear, with a mean threshold of 39.62 ± 9.77 dB HL. Figure 6A shows the disparity between the BC and aided threshold at each frequency. The results for VSB were similar to those for Baha, demonstrating smaller values at 1 kHz and 2 kHz and larger values at lower and higher frequencies. However, one difference between the two was that VSB users often utilized it above the BC threshold at 1 kHz and 2 kHz.

### 3.8. VSB: Functional Gain

Significant FG was observed at 1000 Hz, with FGs at each frequency as follows: 250 Hz, 13.08 ± 17.14 dB HL; 500 Hz, 27.69 ± 17.27 dB HL; 1000 Hz, 38.46 ± 18.30 dB HL; 2000 Hz, 34.23 ± 13.36 dB HL; 4000 Hz, 30.00 ± 15.68 dB HL. The average FG across the four frequencies was 32.60 ± 16.16 dB HL.

### 3.9. VSB: Correlation between Preoperative BC Threshold and Aided Threshold

**Figure 7:**
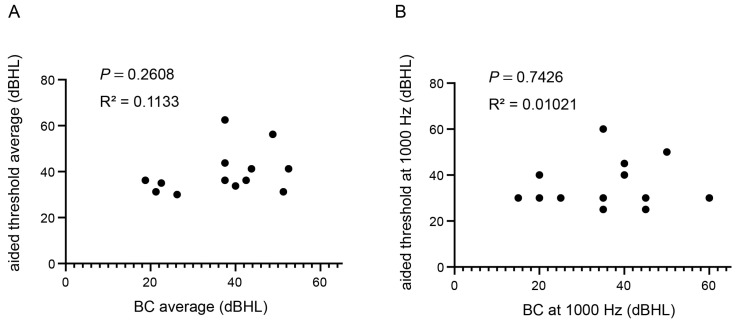
Correlation between preoperative bone-conduction (BC) threshold and aided threshold with Vibrant Soundbridge. (**A**) Mean BC thresholds at 500, 1000, 2000, and 4000 Hz for the better BC threshold were treated as independent variables, while the mean aided threshold was treated as the dependent variable. (**B**) Preoperative BC threshold at 1000 Hz for the better-hearing ear was used as the independent variable and the aided threshold at 1000 Hz was used as the dependent variable.

Linear regression analysis of preoperative BC and aided thresholds was performed to test whether the preoperative BC threshold could predict the effectiveness of VSB. When the mean BC thresholds of 500, 1000, 2000, and 4000 Hz were set as independent variables and the mean aided threshold was set as the dependent variable, no significant correlation was found, with an R^2^ value of 0.113 and a *p*-value of 0.261 (Figure 7A). Unlike the results from Baha, when the preoperative BC threshold of the better-hearing ear at 1000 Hz was set as the independent variable and the aided threshold at 1000 Hz as the dependent variable, no positive correlation was observed, with an R^2^ value of 0.010 and a *p*-value of 0.743 (Figure 7B).

### 3.10. VSB: Word Recognition Score

**Figure 8:**
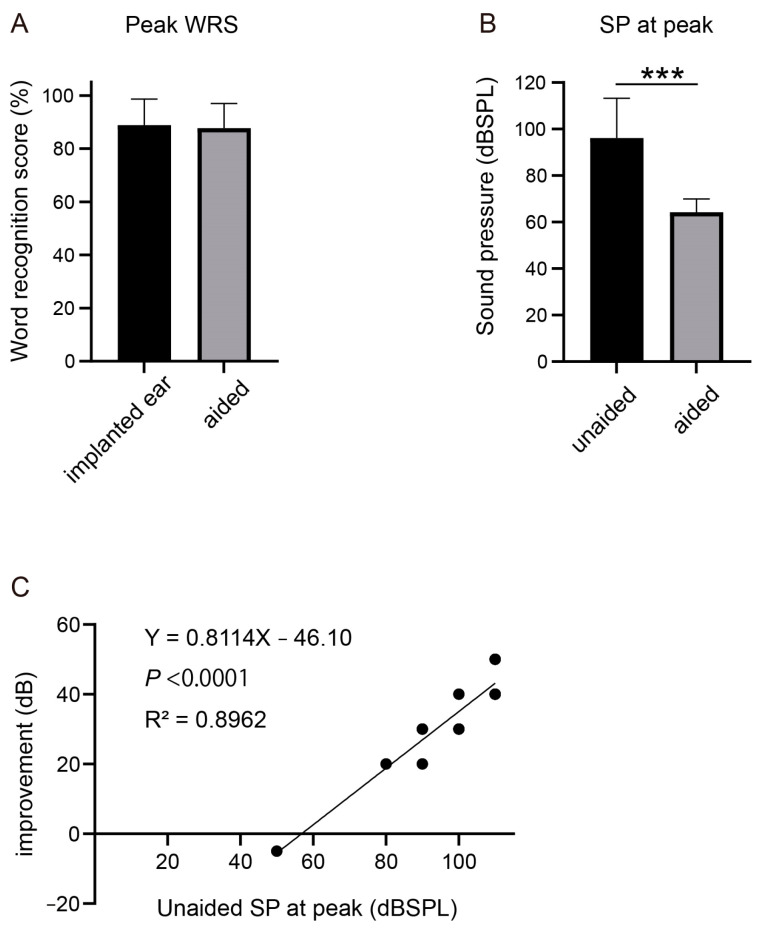
Word recognition score (WRS) compared between unaided and aided condition with Vibrant Soundbridge (VSB). (**A**) Peak WRS in unaided and aided condition with VSB. (**B**) Sound pressure (SP) required to reach the peak WRS in unaided and aided condition. *** *p* < 0.001. (**C**) Correlation between preoperative SP at peak and the improvement of SP at peak.

The peak WRS with VSB did not show improvement compared to that of the better ear preoperatively (Figure 8A). However, similar to the results of Baha, the SP required to reach the peak WRS significantly decreased with VSB (*p* < 0.001) (Figure 8B). To investigate whether this improvement could be predicted preoperatively, we utilized preoperative SP at peak as the independent variable and the improvement in SP at peak as the dependent variable. The resulting R^2^ value was 0.896, and the *p*-value was <0.0001, indicating a positive correlation.

## 4. Discussion

This report assessed the efficacy of BCIs and MEIs as hearing support devices for patients who underwent surgery for refractory otitis media. The average aided thresholds for BCIs and MEIs were 37.34 dB HL and 39.62 dB HL, respectively, which were close to the BC threshold of the better-hearing ear side for BCIs and the BC threshold of the implanted side for MEIs. The FG for BCIs was 51.36 dB on average across the four frequencies, whereas for it was 32.60 dB for MEIs. This study is the first to assess the effect of these devices only in postoperative patients with refractory otitis media. The FG of each device was comparable to that of previous reports assessing the effect of mixed or conductive hearing loss.

Several studies have compared the unaided hearing ability to the aided threshold with Baha. Cooper et al. analyzed patients in four subgroups according to previously used hearing aids (AC or BC hearing aid) and etiology (chronic suppurative otitis media or congenital hearing loss). In all subgroups, the mean sound-field warble-tone thresholds showed significant improvements from unaided to aided with Baha [10]. Béjar-Solar et al. performed Baha implantation in 11 patients with bilateral atresia and reported that the threshold improved from 64 dB in the unaided condition to 19 dB in the Baha-aided condition [14]. Burrell et al. reported the effect of Baha in nine patients with otosclerosis and found that the threshold improved from 49.4 dB in the unaided condition to 30.6 dB in the Baha-aided condition [15]. Compared with these results, the effect of a Baha in postoperative patients with refractory otitis media in this study was considered favorable. The following two factors were considered to be responsible for the large FG obtained in our report compared to other reports: (1) the air-bone gap of the postoperative patients with refractory otitis media was large (44.08 ± 12.17 dB), and (2) the BC threshold on the implanted side of the Baha was better than that on the contralateral side. It is possible that this could be attributed to damage within the inner ear caused by chronic inflammation, and there was a significant difference between the mean BC threshold of 44.62 dB on the implanted side and 31.47 dB on the contralateral side (*p* = 0.013). Since the Baha provides a BC threshold close to that of the better-hearing ear, in cases with a preserved BC threshold of the better-hearing ear, it is possible that a large FG could be obtained.

Several studies have compared auditory outcomes in the unaided condition with those in the aided condition using a VSB. However, only a handful of studies have analyzed the impact of a VSB on hearing with a focus on its etiology. Bernardeschi et al. conducted a study in patients with aplasia (*n* = 3), chronic otitis media (*n* = 18), otosclerosis (*n* = 6), and external auditory meatus stenosis (*n* = 2) after VSB implantation. They reported a noteworthy improvement in the hearing of all patients after VSB activation, without any discernible differences between each disease [16]. The postoperative average pure-tone threshold in the unaided condition was 63 dB (*n* = 24) and that in the aided condition with VSB in the free field was 24 dB (*n* = 22). Henseler et al. also performed long-term observations with VSB (FMT within the round window niche) implantation in 10 patients after subtotal petrosectomy and reported an average FG of 35 dB (range 25–44 dB) [11]. The etiology of hearing loss has not yet been investigated. However, a review conducted by the Australian Medical Services Advisory Committee examined the FG of patients with mixed hearing loss by categorizing them according to the severity of their condition. The results showed that the mild–moderate group had an FG range of 26.17–32.0 dB, while the severe group had an FG range of 34.5–49.3 dB [17]. The mean AC and BC thresholds of patients in this article were 72.21 dB HL and 36.92 dB HL, respectively. Because this group of patients was classified as having severe mixed hearing loss, an FG of 32.6 dB was considered low.

In this study, we examined the correlation between the preoperative and aided BC thresholds. For the Baha, as shown in Figure 3, the preoperative mean BC threshold in the better-hearing ear did not correlate with the mean-aided threshold, while the preoperative 1000 Hz better-hearing side threshold correlated with the 1000 Hz aided threshold. This may be because the aided thresholds of the Baha system were close to the better-hearing BC threshold at 1000 Hz, whereas there were discrepancies at 500 and 4000 Hz. However, the results for VSB users showed no correlation, even at 1000 Hz, as shown in Figure 7. These findings suggest that VSBs can potentially achieve a consistent aided threshold irrespective of preoperative hearing ability. The VSB was specifically designed to address the shortcomings of AC hearing aids such as distortion, occlusion, and acoustic feedback. Similar to AC hearing aids, the VSB has proven to be effective in patients with sensorineural hearing loss [18]. Therefore, it is feasible to attain FG without restrictions on the preoperative threshold. As depicted in Figure 6, VSB users in this study exhibited an aided threshold that exceeded the BC threshold of the patient.

In this study, the WRS was analyzed under both unaided and aided conditions at different SPs. Neither device improved the peak WRS. However, the SP required to reach the peak WRS was reduced to approximately 60 dB HL. This effect may be useful in speech comprehension, particularly at low SPs. Linear regression analysis was performed to determine whether the improvement in SP at peak by the devices correlated with the unaided preoperative SP at peak. Our findings indicate a strong association between higher preoperative SP at peak and the effectiveness of the devices in improving SP at peak. Conversely, it was difficult to achieve a similar effect when the initial peak SP was close to 60 dB HL. This information may be valuable in predicting the indications for surgical implants and their postoperative outcomes.

A limitation of this report is that the small number of cases did not allow us to examine postoperative middle ear conditions separately. The following postoperative conditions of refractory otitis media were encompassed in Table 1 and Table 2: radical cavity, post-canal wall-up surgery, and blind sac closure. A subjective evaluation using a questionnaire should also be considered; however, this was not described in this report. This is a topic for future research.

**Figure 9:**
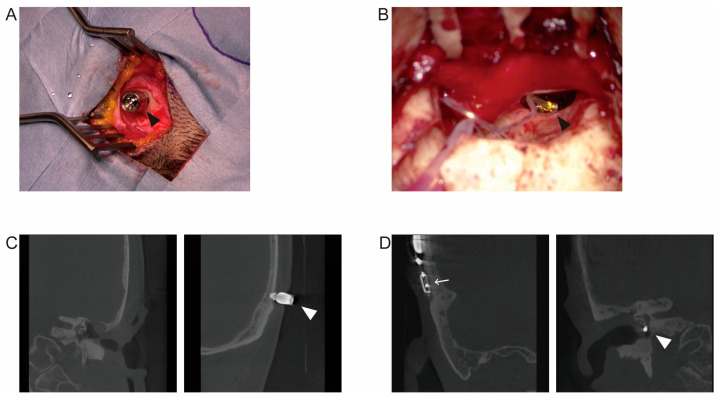
Intraoperative findings and postoperative CT images of implant insertion surgeries. (**A**) Observation during the Baha implant surgery. The arrowhead indicated the implant embedded in the temporal bone and the connected abutment. (**B**) Observation during the Vibrant Soundbridge (VSB) implant surgery. The coil was left in the cavity after a mastoidectomy and the floating mass transducer was installed in the round window niche (indicated by the arrowhead). (**C**) Coronal sections of a CT image following Baha implant surgery on left side. The arrowhead indicates the implant embedded in the temporal bone and the abutment. The abutment was exposed on the skin surface. (**D**) Coronal sections of a CT image following VSB implant surgery on right side. The implant (indicated by the arrow) was fixed to the temporal bone with screws and was buried subcuta-neously. The arrowhead indicates the floating mass transducer left in the round window niche.

In postoperative cases of refractory otitis media, the condition of the middle ear differed from normal and included mastoidectomy and/or removal of the posterior canal wall. However, our findings indicated that both BCIs and MEIs yielded favorable hearing outcomes. In terms of auditory characteristics, MEIs boast unilateral stimulation of the aided ear, which is anticipated to facilitate binaural effects such as summation, head shadow, and squelch. It also confers an advantage for sound localization. Conversely, BCIs stimulate both the implanted and contralateral sides, rendering it arduous to ascertain the direction. However, contralateral stimulation allows the sound originating from the afflicted ear to be perceived via the inner ear of the healthy ear, making it a suitable choice for single-sided deafness [19]. Each of these devices exhibits unique advantages, grounded not only in their auditory characteristics but also in their physical features. Representative intraoperative findings and postoperative CT images from our surgeries with each of these devices are demonstrated in Figure 9. For instance, Baha implantation requires a relatively simple procedure, which entails making a small skin incision and embedding an implant in the skull bone. When using the Baha Connect system, an abutment becomes exposed on the skin surface. On the other hand, VSB consists of a coil and an FMT emanating from an implant affixed to the temporal bone with screws. Ensuring the secure placement of the FMT on the round window niche or the ossicles necessitates a higher degree of surgical precision. Contrary to Baha, no part of the implant is exposed on the skin surface in VSB. Given these distinct characteristics of each device, selecting the appropriate one is paramount, and the presence or absence of infection in the middle ear cavity could be a significant factor. In cases where surgery is performed on the infected ear, a BCI could be suitable. A viable strategy when choosing an MEI involves undertaking preparation surgeries, such as blind sac closure of the external auditory canal or posterior wall reconstruction, to control inflammation before the implantation. Indeed, we performed preparation surgery in three cases and proceeded with the MEI implantation in the second operation (Table 2). Caution must be exercised when considering a VSB implantation for patients with an open cavity, regardless of the presence or absence of chronic inflammation, where the posterior wall of the external auditory canal has been removed. Barbara et al. reported postoperative complications in approximately half of the 21 adult cases where VSB was implanted in an open cavity, with lead exposure (23.8%) being the most common complication [20]. While the report suggested that corrective surgery using large cartilage grafts was effective, preparation surgery can be anticipated to be effective for open cavities. It also should be noted that assessing the brain or lateral skull with magnetic resonance imaging can be more challenging when using a VSB, as the generated artifact can interfere with the accuracy of the results. These characteristics of each device, in conjunction with those obtained in the present investigation, could help in selecting an appropriate hearing support method for patients who have undergone middle ear surgery and in predicting the effectiveness of the selected device.

## 5. Conclusions

This study investigated the hearing outcomes in patients who underwent MEI or BCI implantation after surgery for refractory otitis media. Furthermore, we examined the preoperative parameters that may serve as predictors of the effectiveness of hearing outcomes after implantation. The findings of this study have significant implications for selecting suitable hearing devices for postoperative patients with refractory otitis media and predicting the effect of such devices.

## Figures and Tables

**Table 1:** Summary of clinical features in Baha-implanted subjects.

Baha				PTA Average ^a^ on Aided Side		PTA Average on Contralateral Side			
Subject ID	Gender	Age (year)	Implanted Side	AC ^b^	BC ^c^	AC	BC	Pathology	Processor
1	F	78	L	87.5	43.75	72.5	43.75	radical cavity	Baha5
2	M	72	L	52.5	13.75	110	66.25	radical cavity	Baha5
3	F	56	R	110	66.25	50	33.75	radical cavity	Baha5
4	M	54	R	98.75	47.5	52.5	20	post CWU ^d^	Baha5
5	M	60	R	110	66.25	46.25	17.5	bllind sac closure	Baha5
6	M	68	L	62.5	40	106.25	65	radical cavity	Baha5
7	F	21	L	58.75	22.5	46.25	22.5	post CWU	Baha5
8	M	63	R	63.75	23.75	70	31.25	post CWU	Baha5
9	F	80	L	96.25	63.75	66.25	30	post CWU	Baha5
10	M	67	R	93.75	62.5	37.5	26.25	bllind sac closure	Baha5
11	F	76	L	127.5	66.25	72.5	31.25	radical cavity	Baha5
12	M	71	L	127.5	66.25	45	28.75	radical cavity	Baha5
13	M	68	L	87.5	37.5	22.5	22.5	post CWU	Baha5
14	M	72	R	116.25	66.25	41.25	23.75	post CWU	Baha5
15	F	67	R	97.5	40	76.25	32.5	radical cavity	Baha5
16	M	79	R	61.25	25	85	56.25	post CWU	Baha5
17	M	71	R	85	32.5	37.5	27.5	bllind sac closure	Baha5
18	M	62	L	106.25	58.75	36.25	32.5	post CWU	Baha5
19	F	54	R	40	22.5	26.25	13.75	post CWU	Baha5
20	M	52	L	96.25	41.25	50	26.25	bllind sac closure	Baha5
21	F	74	R	52.5	22.5	58.75	30	post CWU	Baha5
22	F	72	R	98.75	48.75	35	33.75	radical cavity	Baha5 Power
23	F	64	R	110	48.75	23.75	8.75	bllind sac closure	Baha5

^a^ Pure tone audiometry average (dB HL) of 500, 1000, 2000, and 4000 Hz. ^b^ Air-conduction threshold. ^c^ Bone-conduction threshold. ^d^ Canal wall up surgery.

**Table 2:** Summary of clinical features in Vibrant Soundbridge-implanted subjects.

VSB			PTA Average ^a^ on Aided Side		PTA Average on Contralateral Side				
Subject ID	Gender	Age (year)	AC ^b^	BC ^c^	AC	BC	Pathology	Preparation Surgery	FMT ^d^
1	F	69	65	37.5	67.5	40	post CWU ^e^	N/A	RW
2	M	61	70	42.5	72.5	46.25	post CWU	N/A	RW
3	F	60	66.25	26.25	55	22.5	radical cavity	blind sac	RW
4	F	73	67.5	37.5	48.75	27.5	radical cavity	N/A	RW
5	F	75	81.25	43.75	87.5	47.5	radical cavity	blind sac	RW
6	F	82	67.5	22.5	103.75	63.75	radical cavity	N/A	RW
7	F	71	95	48.75	53.75	50	radical cavity	N/A	Stapes
8	M	75	70	51.25	52.5	36.25	post CWU	N/A	RW
9	F	67	55	21.25	61.25	23.75	radical cavity	CWR ^f^	RW
10	F	59	65	37.5	57.5	32.5	post CWU	N/A	RW
11	F	31	62.5	18.75	60	20	post CWU	N/A	RW
12	F	67	98.75	52.5	91.25	60	post CWU	N/A	RW
13	M	64	75	40	30	27.5	post CWU	N/A	RW

^a^ Pure tone audiometry average (dB HL) of 500, 1000, 2000, and 4000 Hz. ^b^ Air-conduction threshold. ^c^ Bone-conduction threshold. ^d^ Floating mass transducer. ^e^ Canal wall up surgery. ^f^ Canal wall reconstruction.

## Data Availability

Data are available upon reasonable request to the corresponding authors.

## References

[1] Nishimura T., Hosoi H., Sugiuchi T., Matsumoto N., Nishiyama T., Kenichi T., Sugimoto S., Yazama H., Sato T., Komori M. (2021). Cartilage conduction hearing aid fitting in clinical practice. J. Am. Acad. Audiol..

[2] Mudry A., Tjellström A. (2011). Historical background of bone conduction hearing devices and bone conduction hearing aids. Adv. Oto-Rhino-Laryngol..

[3] Colquitt J.L., Jones J., Harris P., Loveman E., Bird A., Clegg A.J., Baguley D.M., Proops D.W., Mitchell T.E., Sheehan P.Z. (2011). Bone-anchored hearing aids (BAHAs) for people who are bilaterally deaf: A systematic review and economic evaluation. Health Technol. Assess..

[4] Danhauer J.L., Johnson C.E., Mixon M. (2010). Does the evidence support use of the Baha implant system (Baha) in patients with congenital unilateral aural atresia?. J. Am. Acad. Audiol..

[5] Johnson C.E., Danhauer J.L., Reith A.C., Latiolais L.N. (2006). A systematic review of the nonacoustic benefits of bone-anchored hearing AIDS. Ear Hear..

[6] Secretariat M.A. (2002). Bone anchored hearing aid: An evidence-based analysis. Ont. Health Technol. Assess. Ser..

[7] Ernst A., Todt I., Wagner J. (2016). Safety and effectiveness of the Vibrant Soundbridge in treating conductive and mixed hearing loss: A systematic review. Laryngoscope.

[8] Klein K., Nardelli A., Stafinski T. (2012). A systematic review of the safety and effectiveness of fully implantable middle ear hearing devices: The carina and esteem systems. Otol. Neurotol..

[9] Verhaert N., Desloovere C., Wouters J. (2013). Acoustic hearing implants for mixed hearing loss: A systematic review. Otol. Neurotol..

[10] Cooper H.R., Burrell S.P., Powell R.H., Proops D.W., Bickerton J.A. (1996). The Birmingham bone anchored hearing aid programme: Referrals, selection, rehabilitation, philosophy and adult results. J. Laryngol. Otol. Suppl..

[11] Henseler M.A., Polanski J.F., Schlegel C., Linder T. (2014). Active middle ear implants in patients undergoing subtotal petrosectomy: Long-term follow-up. Otol. Neurotol..

[12] Iwasaki S., Usami S., Takahashi H., Kanda Y., Tono T., Doi K., Kumakawa K., Gyo K., Naito Y., Kanzaki S. (2017). Round Window Application of an Active Middle Ear Implant: A Comparison with Hearing Aid Usage in Japan. Otol. Neurotol..

[13] Japan Otological Society (2019). Indication Criteria for Bone-Anchored Hearing Aids (Baha^®^ System). https://www.otology.gr.jp/.

[14] Béjar-Solar I., Rosete M., de Jesus Madrazo M., Baltierra C. (2000). Percutaneous bone-anchored hearing aids at a pediatric institution. Otolaryngol. Head Neck Surg..

[15] Burrell S.P., Cooper H.C., Proops D.W. (1996). The bone anchored hearing aid—The third option for otosclerosis. J. Laryngol. Otol. Suppl..

[16] Bernardeschi D., Hoffman C., Benchaa T., Labassi S., Beliaeff M., Sterkers O., Grayeli A.B. (2011). Functional results of Vibrant Soundbridge middle ear implants in conductive and mixed hearing losses. Audiol. Neurootol..

[17] The Medical Services Advisory Committee (2010). Middle Ear Implant for Sensorineural, Conductive and Mixed Hearing Losses.

[18] Kahue C.N., Carlson M.L., Daugherty J.A., Haynes D.S., Glasscock M.E. (2014). Middle ear implants for rehabilitation of sensorineural hearing loss: A systematic review of FDA approved devices. Otol. Neurotol..

[19] Kim G., Ju H.M., Lee S.H., Kim H.S., Kwon J.A., Seo Y.J. (2017). Efficacy of bone-anchored hearing Aids in single-sided deafness: A systematic review. Otol. Neurotol..

[20] Barbara M., Volpini L., Covelli E., Romeo M., Filippi C., Monini S. (2019). Complications after round window vibroplasty. Eur. Arch. Otorhinolaryngol..

